# Assessment of the Effect of Remediation Strategies on the Environmental Quality of Aquaculture Ponds in Marilao and Meycauayan, Bulacan, Philippines

**DOI:** 10.5696/2156-9614-8.20.181205

**Published:** 2018-12-03

**Authors:** John Vincent R. Pleto, Mark Dondi M. Arboleda, Jessica F. Simbahan, Veronica P. Migo

**Affiliations:** 1 Environmental Biology Division, Institute of Biological Sciences, University of the Philippines, Los Baños, Philippines; 2 School of Environmental Science and Management, University of the Philippines, Los Baños, Philippines; 3 Institute of Biology, College of Science, University of the Philippines, Diliman, Philippines; 4 Department of Chemical Engineering, College of Engineering and Agro-Industrial Technology, University of the Philippines, Los Baños, Philippines

**Keywords:** Marilao-Meycauayan-Obando river system, phytoremediation, probiotics, zeolite

## Abstract

**Background.:**

Water quality in the Marilao-Meycauayan-Obando river system (MMORS) of Bulacan, the Philippines, is of great concern due to the pollution load from local industries. The river system is currently used as a source of water for the aquaculture industry in Bulacan.

**Objectives.:**

In order to address organic and heavy metal pollution, several remediation strategies were tested in aquaculture ponds along the river system. Strategies such as phytoremediation (vetiver grass pontoons), application of probiotics and zeolite (with filtration as pre-treatment) were utilized in ponds to decrease or remove toxic pollutants in water and sediments.

**Methods.:**

Two sites were chosen as the pilot remediation sites – ponds in Barangay Nagbalon, Marilao and Barangay Liputan, Meycauayan, Bulacan. Pond bottom preparation was done to improve the condition of the pond bottom sediments before stocking by adding zeolite. Physicochemical parameters of water such as dissolved oxygen (DO), temperature, pH, salinity, ammonia, phosphate, biochemical oxygen demand (BOD) and chemical oxygen demand (COD) were monitored throughout the culture period. Heavy metals in sediments and fish were monitored. Fish parameters such as average body weight and feed conversion ratio were determined.

**Results.:**

The DO levels were below recommended levels in the morning and reached a supersaturated level in the afternoon. Ammonia and COD levels were above recommended limits. A decreasing trend was observed for ammonia levels in treatment ponds. In terms of the growth of milkfish, the pond with probiotics showed the highest growth and better feed conversion ratio in Nagbalon and in the phytoremediation pond in Liputan. Percentage survival of milkfish was much higher at Liputan. Copper, chromium, lead and manganese were detected in pond sediments. After application of zeolite, there was a decrease in lead levels throughout the culture period.

**Conclusions.:**

The different remediation studies were compared in terms of cost, effectivity and application and phytoremediation (vetiver grass pontoons) was determined to be the most cost-effective remediation strategy.

**Competing Interests.:**

The authors declare no competing financial interests.

## Introduction

Pollution in the aquatic environment is often a direct or indirect result of human activities and can result in damage to living resources, hazards to human health, a burden to aquatic activities and impairment of water quality. Water quality in the Marilao-Meycauayan-Obando river system (MMORS) is of great concern as formal and informal industries utilizing toxic heavy metals located along the river system discharge their untreated wastewater into the river.^1^ Heavy metal and organic pollution are severe on the river system and have caused environmental resource degradation and pose numerous public health risks. In order to address severe water pollution in the river system, several remediation strategies have been utilized. Bioremediation uses plants and/or microorganisms such as bacteria, protozoa and fungi to degrade contaminants in soil and groundwater into less toxic or non-toxic compounds.^2^,^3^ Bioremediation is a low cost and eco-friendly technology and involves not only the degradation of pollutants, but is also often able to remove pollutants from the environment without further degradation.^4^

The present study evaluates the baseline physicochemical characteristics of aquaculture ponds of Nagbalon and Liputan. It aims to assess the performance of different remediation strategies in terms of the physicochemical changes in water and the heavy metal content of water and sediments along with the growth performance of stocked milkfish. In addition, it aims to identify the most effective pollution mitigation strategy for use in aquaculture ponds in Bulacan.

## Methods

Fishponds using water from the Marilao and Meycauayan River of the MMORS Meycauayan-Marilao-Obando river system were the focus of the present study. This study site was chosen because the water coming from the Marilao and Meycauayan River is said to be contaminated with different heavy metals and is heavily polluted with organic and inorganic matter.

**Abbreviations**
*ABW*: Average body weight
*BOD*: Biochemical oxygen demand
*FCR*: Feed conversion ratio
*COD*: Chemical oxygen demand
*DO*: Dissolved oxygen
*XRF*: X-ray fluorescence

The two sampling areas were located at Barangay Nagbalon, Marilao, Bulacan Province and Barangay Liputan, Meycauayan, Bulacan Province, the Phillippines. The Nagbalon site has a total of four ponds (3 treatment and 1 control) while the Liputan site has 3 ponds (2 treatment and 1 control).

Table 1 shows the pond treatments, geographic coordinates and the physical characteristics of the studied ponds.

**Table 1:**
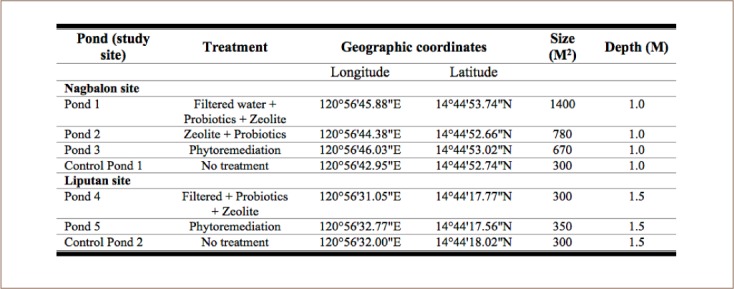
Geographic Coordinates, Physical Characteristics and Pond Treatments

The filtration pond, which is composed of pipes that transfer water to the test ponds, was established to filter the water coming from the river and supply water to Pond 1 at the Nagbalon site and Pond 4 at the Liputan site. It is composed of gravel, pebbles and sand that filters the incoming water from the river. There is a perforated pipe manifold at the bottom to transfer the water to the experimental pond.

The plant used in the present study was vetiver grass (Chrysopogon sp) purchased from Vetiver Farms, the Philippines. The vetiver grass was set up in 1 × 1 m square bamboo pontoons. Twelve or more vetiver mats were installed on each bamboo pontoon. The bamboo pontoons with vetiver grass were floated on the ponds. These grasses were later collected and utilized for heavy metals analysis.

Zeolites are hydrated aluminosilicate minerals that contain alkali and alkaline earth materials. These are used as commercial adsorbents and catalysts. They were applied after pond preparation, which involved removing the upper layer of the sediments and scattering the zeolite powder throughout the pond sediment before introduction of water.

The probiotic used in the present study was purchased from CP (Charoen Phokphand) Feeds in Samal, Bataan, the Philippines, consisted of Super biotic^®^ and pH fixer. Super biotic was applied during pond preparation and the pH fixer was applied after 2 months of culture. The super biotic was applied at a dosage of 3 L per ha. and the pH Fixer^®^ at a dosage of 150 g per ha. It was mixed with molasses with water at a rate of 500 ml molasses and 100 L of water for every 1 L Super biotic^®^ and was incubated for 24 hours in a drum before application to the ponds. The mixture was dispersed throughout the pond water.

### Baseline characterization of ponds

A baseline characterization of ponds was done to determine the condition of the ponds at both sites. Water quality parameters include dissolved oxygen, pH, temperature, salinity, chemical oxygen demand, biochemical oxygen demand, ammonia and phosphates. Heavy metals were determined by collecting water and sediment samples from the ponds and submitted to the laboratory for testing.

### Water quality pond monitoring

*In situ* and *ex situ* procedures were followed in the determination of water quality parameters. Parameters analyzed *in situ* included dissolved oxygen (DO), pH, temperature and salinity. *Ex situ* determination of parameters included ammonia (NH_3_), phosphate (PO_4_), biochemical oxygen demand (BOD), and chemical oxygen demand (COD).

Water quality monitoring was performed daily on the following parameters: DO, temperature, pH, and salinity. *Ex situ* parameters such as NH_3_ and PO_4_ were monitored on a weekly basis. Monthly monitoring of parameters included chemical COD and BOD. *In situ* monitoring was done around 5:30 to 8:30 in the morning and 13:00 to 16:00 in the afternoon. Water samples were collected from four random sampling points at each pond and pooled into two 1-L polyethylene bottles. The remaining parameters such as BOD, COD and heavy metals (arsenic (As), cadmium (Cd), copper (Cu), and lead (Pb)) were analyzed monthly in the laboratory. Table 2 shows the analytical methods used in determining water quality.

**Table 2:**
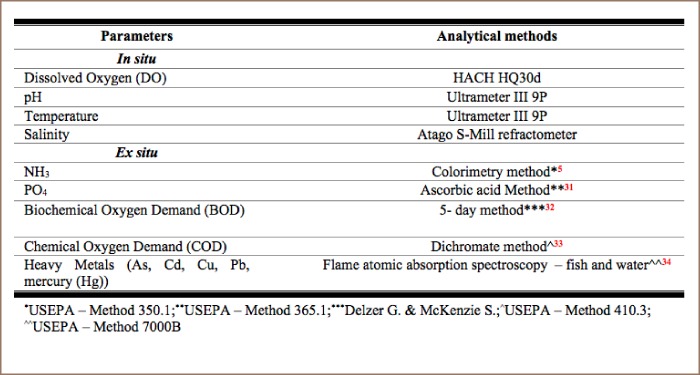
Analytical Methods Used for Water Quality Parameters

Ponds were monitored for 17 weeks, or 4 months, which is the prescribed time for milkfish culture.

### Sediment heavy metals analysis and monitoring

Baseline sediment analysis was done to determine the changes brought about by the zeolite. Monthly sediment sampling from the different ponds was done to determine the levels of heavy metals (As, Cd, Cu, chromium (Cr), Hg, Pb, manganese (Mn), nickel (Ni) and zinc (Zn)) in each pond. Sediment samples were collected from four random sites on each pond. The sediments were pooled for heavy metal analysis. Sediment samples were air dried and ground into fine powder using a mortar and pestle. The sediments were analyzed using a Niton X-ray fluorescence (XRF) spectrophotometer. The XRF spectrophotometer is widely accepted for environmental use in field screening of heavy metals.^6^

### Fish sampling and analysis

Fish sampling was done monthly to determine the levels of heavy metals in each pond. Representative fish samples for each pond were collected to determine size and weight. The average body weight (ABW) was obtained and feed conversion ratio (FCR) was computed. The fish samples were harvested for heavy metal analysis using the flame atomic absorption spectrophotometry method.

### Statistical analysis

Analysis of covariance was done to compare the difference between ponds (spatial) and the temporal changes in terms of the physicochemical parameters during the culture period. The Pearson correlation coefficient was used to determine the relationship of the physicochemical and fish parameters. The data were analyzed using the Statistical Analysis System (SAS) software package.

## Results

Baseline characterization of ponds was done to determine the impact of the different remediation strategies on water and sediment quality. The baseline physicochemical characteristics of both ponds and the recommended levels at Nagbalon and Liputan are shown in Tables 3 and 4.

**Table 3:**
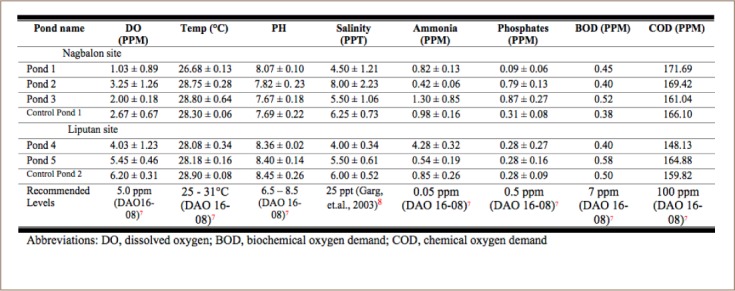
Baseline Characterization of Ponds Before Pond Preparation (November 2014)

**Table 4:**
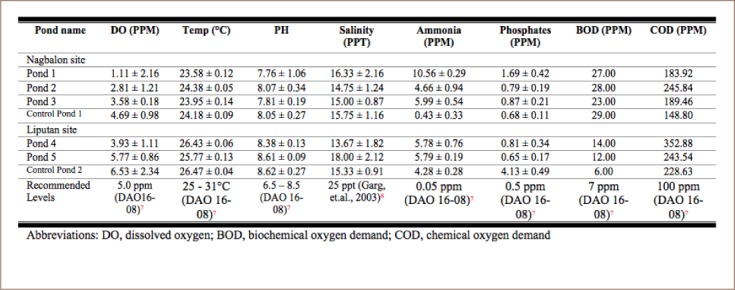
Baseline Characterization of Ponds After Pond Preparation (February 2015)

In the present study, the dissolved oxygen at both sites before and after pond preparation was below the recommended limit of 5.0 ppm. The water temperature was relatively low at both sites before and after pond preparation. The pH level was slightly alkaline. Salinity levels increased significantly after pond preparation. The ammonia levels exceeded the permissible limit before and after pond preparation. The phosphate and BOD levels before pond preparation were below the safe limits, but exceeded safe limits after pond preparation. The COD levels were above the recommended limit before and after pond preparation.

Table 5 shows the heavy metal concentrations of pond water before and after pond preparation. In the present study, levels of heavy metals in water did not exceed recommended limits. The only detected metal which exceeded the recommended level based on DAO16-08 was Cu (30.0 ppb) in Pond 2 after pond preparation.

**Table 5:**
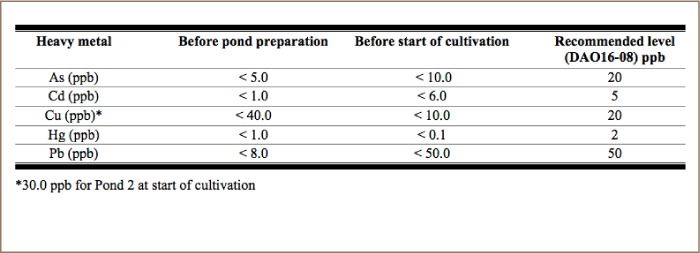
Heavy Metals Concentrations in Pond Water Before Pond Preparation and at the Start of Cultivation for Ponds at Nagbalon and Liputan

### Water quality pond monitoring

**Figures 1a–1d:**
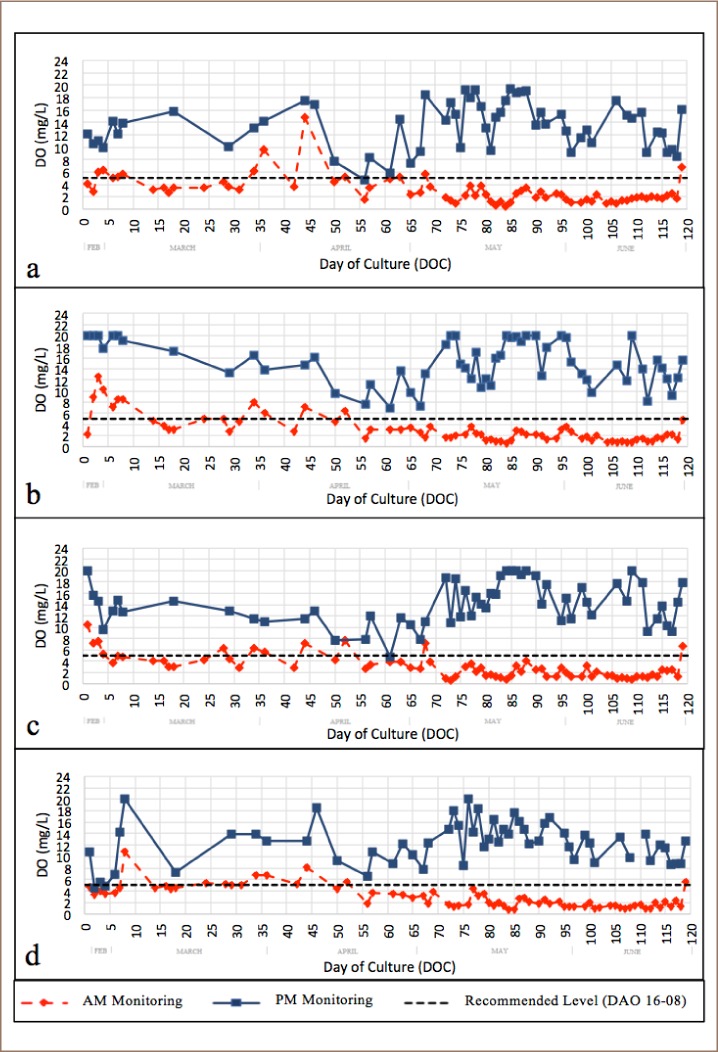
Oxygen levels in ponds at the Nagbalon site in the morning and afternoon. (a) Pond 1 (filtered + probiotics + zeolite); (b) Pond 2 (probiotics + zeolite); (c) Pond 3 (phytoremediation) and (d) Control Pond 1

**Figures 2a–2c:**
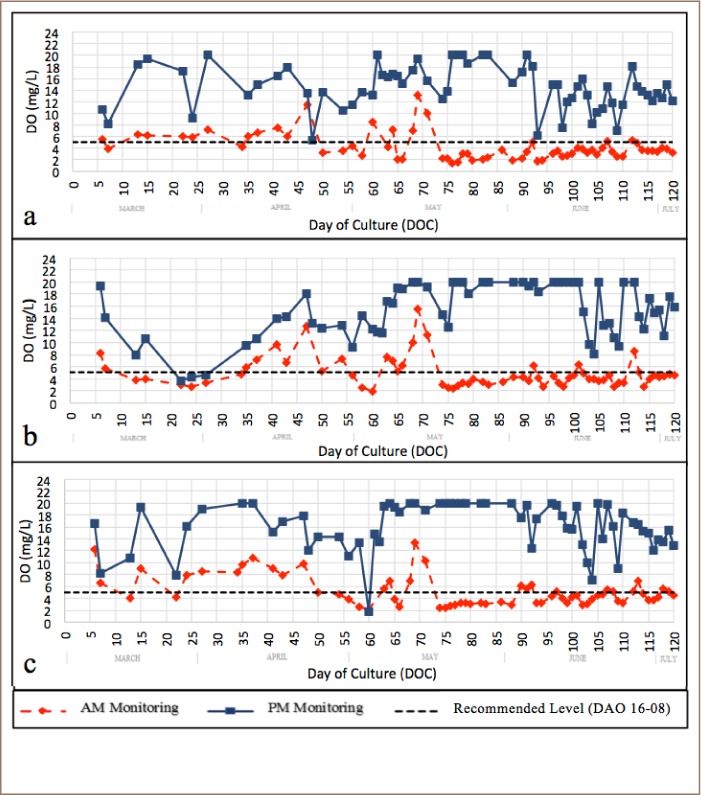
Oxygen levels in ponds at the Liputan site in the morning and afternoon monitoring. (a) Pond 4 (filtered + probiotics + zeolite); (b) Pond 5 (phytoremediation) and (c) Control Pond 2

The dissolved oxygen levels for the entire culture period for both study sites are shown in Figures 1 and 2. In general, the DO level in the morning for all ponds at both sites was generally less than the recommended limit of 5.0 ppm. The highest and lowest recorded DO level was 20 ppm and 0.5 ppm, respectively. The highest and lowest DO levels occurred most frequently during the month of May.

Daily temperature levels for most of the ponds were within recommended limits (25 - 31°C) based on the DAO 16-08 standard for Water Quality Guidelines and General Effluent Standards of 2016.^7^ Temperatures were within the tolerable range, although there were days, especially in the afternoon, when the temperature reached a maximum of 35°C.

The pH of natural water bodies is greatly influenced by the concentration of carbon dioxide, which is an acidic gas.^9^ Based on the Water Quality Guidelines and General Effluent Standard set by the Department of Environment and Natural Resources,^7^ the pH for class C water should be in the range of 6.5 – 8.5. In general, pH levels increased in the afternoon for all study ponds. However, ponds at Liputan (Pond 5 and Control Pond 2) exceeded pH 8.5, which could be stressful for fishes.

**Figure 3:**
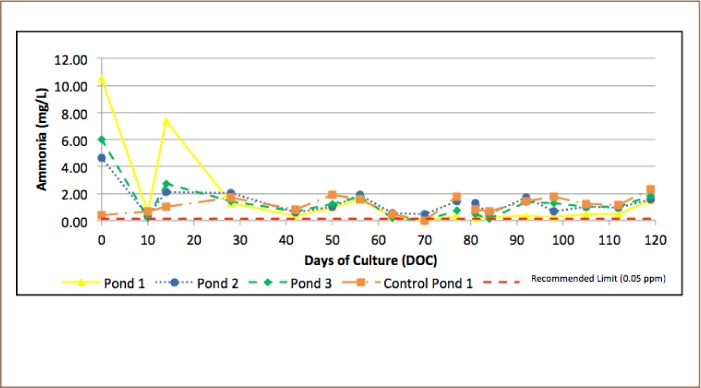
Ammonia levels of ponds at the Nagbalon study site over the entire culture period

**Figure 4:**
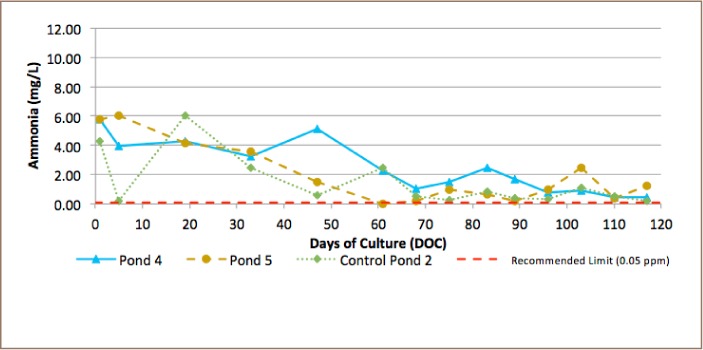
Ammonia levels of ponds at the Liputan study site over the entire culture period

Data on ammonia levels of ponds at both study sites are shown in Figures 3 and 4. All ponds at both sites exceeded the recommended limit of 0.05 ppm.^7^ Most of the ponds had very high ammonia levels at the start, as the reduction of ammonia in water bodies is not very efficient. However, a decreasing trend in ammonia level was observed at all treatment ponds. Comparing all ponds, the phytoremediation ponds had relatively lower ammonia levels compared to other ponds.

The salinity levels in all ponds at both sites were relatively high. Salinity ranged from 15 – 29 ppt for all ponds. Salinity in the morning and the afternoon for all ponds in both Nagbalon and Liputan increased from February to March and then decreased to June and July. The salinity level in the morning was much higher than in the afternoon. The phosphate levels on all ponds for both sites exceeded the recommended limit of DAO 16-08 of 0.5 ppm throughout the culture period. However, a 1.0 ppm level of phosphate is good for plankton production, which serves as fish food. The COD level of all ponds throughout the culture period exceeded the recommended limit set by Department of Environment and Natural Resources, Philippines of 100 ppm.^7^ However, based on the results, the phytoremediation pond at both sites had relatively lower COD compared to other ponds. The BOD levels of all ponds exceeded the recommended level (7 ppm – DAO 16-08) at baseline and the 90th day of culture. All the ponds with treatments had relatively lower BOD compared to the control ponds at both sites. Based on the data obtained, BOD is relatively low and organic matter is present and being decomposed by bacteria.

Table 6 show the Cu content of water at the Nagbalon and Liputan sites. During the 1st and 2nd months of monitoring, small amounts of Cu were detected in ponds at Nagbalon. On the 3rd and 4th months of monitoring, Cu levels were below the detection limit of 0.01 ppm. During the 1st and 2nd month of cultivation, Cu levels in ponds at the Nagbalon site were greater than or equal to the recommended level of 0.02 ppm.^7^ For the other heavy metals such as As, Cd, Pb and Hg, levels at the Nagbalon and Liputan ponds were below detection limits.

**Table 6:**
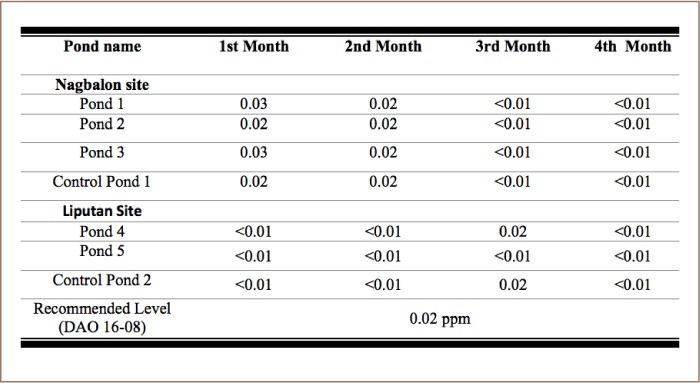
Copper Concentrations (ppm) in Water of Ponds at Nagbalon and Liputan

### Statistical analysis of water quality parameters

One-way analysis of variance for the Nagbalon site showed that pH differed significantly between ponds during the morning, while DO and salinity differed significantly between ponds in the afternoon. For the temporal analysis, DO, temperature and pH showed significant differences between morning and afternoon. A significant difference was observed for temperature between the morning and afternoon.

For the Liputan site, significant differences between ponds were observed for pH in the morning. For the temporal analysis, DO and temperature showed significant differences between morning and afternoon monitoring times.

Temperature showed a significant difference for the afternoon. For the other parameters, ammonia showed a significant difference over time for the Liputan site.

### Fish growth determination and heavy metals analysis

Table 7 shows the monthly average body weight and feed conversion ratio of milkfish for both sites.

**Table 7:**
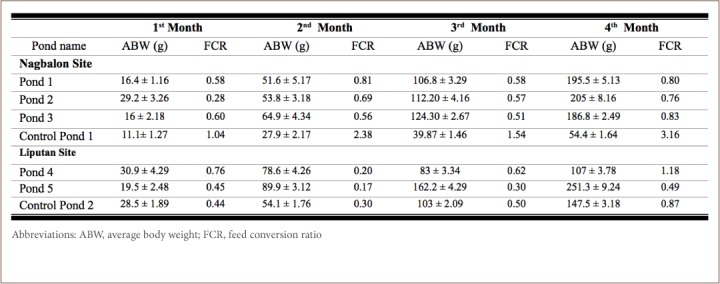
Average Body Weight and Feed Conversion Ratio of Milkfish Across Ponds

The initial body weight of stocked milkfish fingerlings were 4.4 g for Nagbalon and 5.2 g for the Liputan site. After a month of culture, the highest average body weight of milkfish occurred at ponds with probiotics (Pond 2 for Nagbalon and Pond 4 for Liputan). When the phytoremediation setups for both sites were installed, there was a significant increase in the average body weight of milkfish, as reflected in the 2nd and 3rd month of sampling. The phytoremediation functioned as an additional shade and shelter for fishes and helped them survive and grow. In addition, ammonia levels were relatively low when the vetiver grass pontoons were present in the pond water. During the harvest period, the ponds with probiotics showed the highest average body weight of milkfish for the Nagbalon site, while the pond with phytoremediation showed the highest average body weight for the Liputan site.

After a month of milkfish cultivation, the FCR was improved in ponds with probiotics at the Nagbalon site. Moreover, after two and three months of cultivation, phytoremediation showed a better FCR. For the Liputan site, milkfish reared in the phytoremediation pond showed a better FCR throughout the culture period.

Table 8 summarizes the results of the harvest and the percentage survival of milkfishes for ponds at the Nagbalon and Liputan sites.

**Table 8:**
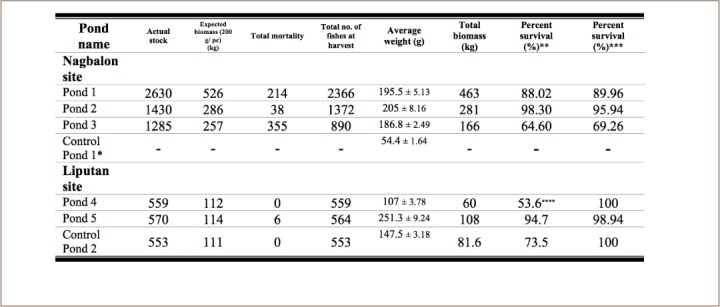
Summary of the Harvest and Percentage Survival of Milkfish in Ponds Across Study Sites

The average weight of milkfish in Nagbalon was found to be profitable. The percentage survival (based on biomass) was highest in Pond 2 at 98.30% and the lowest in Pond 3 (phytoremediation, 64.60%) due to fish mortality. For the Liputan site, Pond 5 (phytoremediation) showed the highest average weight of 251.3 g, while Pond 4 (filtered + probiotics + zeolite) showed an average weight of only 107 g. The percentage survival (based on biomass) was highest for Pond 5 at 94.7% and lowest for Pond 4 at only 53.6%. However, based on fish survival, the ponds at Liputan had a very high percentage survival compared to Nagbalon. However, Pond 4 in Liputan had the lowest biomass.

### Correlation analysis of water quality to growth parameters

Dissolved oxygen, temperature and ammonia had a significantly moderate to strong negative relationship with fish weight. These parameters affect the rate of growth of fishes. For the Liputan site, temperature had a significantly strong positive relationship with fish weight. Ammonia had a significantly strong negative relationship with growth parameters.

### Sediment heavy metal analysis

Table 9 summarizes the heavy metal content of sediments of the different ponds before the application of zeolites.

**Table 9:**
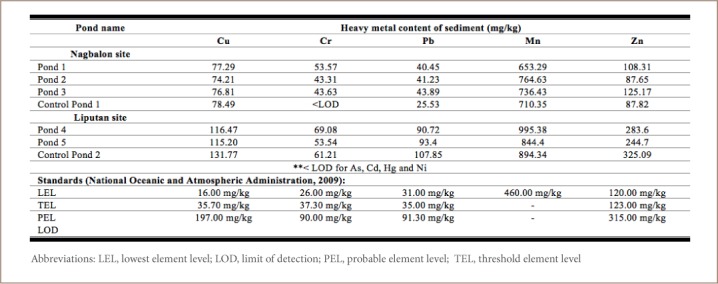
Heavy Metal Content of Sediments Before Application of Zeolite

Treatment of sediment bottom was performed by adding zeolite. These are microporous, aluminosilicate minerals commonly used as commercial adsorbents and catalysts.

The levels of Cu, Cr, Pb and Mn were above the threshold effect level (TEL), but still below the probable effect level (PEL). Arsenic, Cd, Hg and Ni were below the detection limit of the XRF. The TEL is the lower value which represents the concentration below which adverse biological effects are expected to rarely occur. On the other hand, the PEL defines the level above which adverse effects are expected to occur frequently.^10^

Monitoring of heavy metal sediments was performed every month throughout the culture period. Tables 10 – 12 show the monthly heavy metal level of Cu, Cr and Pb.

**Table 10:**
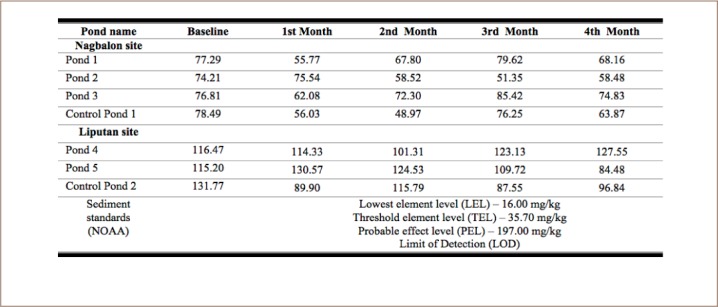
Copper Level (ppb) of Pond Sediments Across Study Sites

**Table 11:**
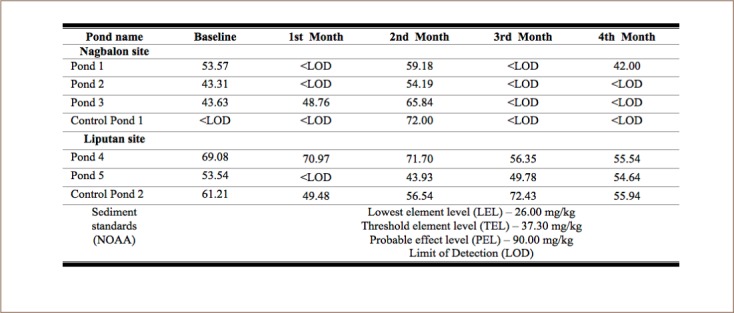
Chromium Level (ppb) of Pond Sediments Across Study Sites

**Table 12:**
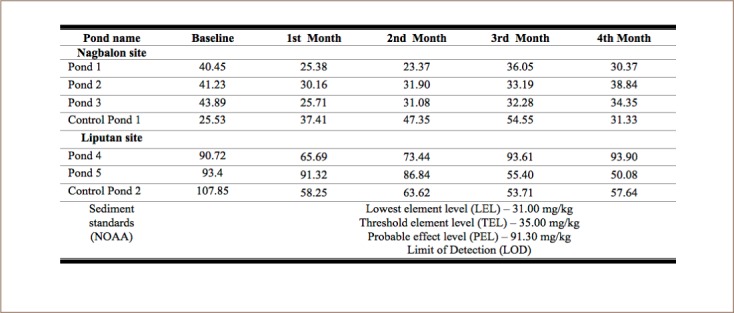
Lead Level (ppb) of Pond Sediments Across Study Sites

Overall, the copper concentration of all ponds exceeded the threshold element level of 31.60 ppm.^11^ When values exceed the TEL, there is a probability that the sediments are toxic to fishes. In the present study, the level did not reach the probable effect level of 197.00 ppm. The Cu concentration of sediments at Liputan site was much higher than at the Nagbalon site. The total Cu concentration of sediments in Pond 5 showed a decreasing trend throughout the culture period. There is a probability that sediments in Liputan ponds were toxic to the milkfish.

Chromium was detected in the sediments for both Nagbalon and Liputan sites. The Cr level at the Liputan site exceeded the threshold element level set by the National Oceanic and Atmospheric Administation. Thus, Cr is likely at elevated and toxic levels. Sediments from both sites were found to contain Pb. Manganese and Zn were present in all ponds, but are not generally harmful to aquatic organisms. Arsenic, Cd and Hg were not detected in sediments at either site. They were below the limit of detection of the XRF.

## Discussion

Dissolved oxygen below the recommended level of 5.00 ppm is stressful for fish and other aquatic life. Fish start to gasp for air on the water surface, which is an indication of a very low DO level. The typical diurnal pattern of dissolved oxygen is very low at dawn (around 5:00 AM), with the highest peak at dusk (around 1:00 PM).^12^ This suggests that DO has been used up at night by the process of respiration, while carbon dioxide has been released. By daytime, DO levels start to increase as photosynthesis takes place in the pond by the production of oxygen by phytoplankton. During the summer months, pond stratification affects the level of DO. There is a high level of dissolved oxygen in the upper layer, where warmer temperatures and phytoplankton growth indicate good water quality, while in the lower layer, there is very low DO and poor phytoplankton growth, indicating poor water quality.^13^ Seasonal stratification occurs, resulting in temperature-dependent density. As water temperature increases, density decreases. Thus, the warm water will remain at the surface, forming the epilimnion, while the denser, cooler water sinks to the bottom forming the hypolimnion. The layer of rapid temperature change separating the two layers is called the thermocline.^14^ Thermocline is the depth at which water temperature begins to decline.^15^ A phenomenon called oxygen supersaturation occurs on ponds every afternoon. This could be detrimental for fishes because when oxygen levels are higher than the saturation level, gas embolisms or trapping of gas bubbles in the blood stream takes place, which could be fatal to fish. Moreover, oxygen can become lethal in some cases when levels are too high, and act as a metabolism depressant. It also inhibits the activity of the respiratory enzyme succinic dehydrogenase.^16^ Supersaturated dissolved oxygen occurs when oxygen is produced by algae more quickly than it can escape into the atmosphere. Another factor of supersaturated DO is the rapid tumbling or movement of water due to wind and fish movement which increases the rate of diffusion.^17^ This supersaturated DO usually lasts for a short period of time, but can be harmful to fish. Rapid aeration and photosynthesis coupled with higher water temperatures are the primary contributors to DO supersaturation. During the process of photosynthesis of algae, which is naturally present in water, oxygen is produced as a waste product and adds to the DO concentration in ponds.^18^

During the day, pond waters often warm up to temperatures higher than those of flowing water in the river. Temperature affects metabolism and physiology and ultimately affects the production of cultured fish. Higher temperatures increase the rate of biochemical activity of microbiota and plant respiratory rates, causing an increase in oxygen demand that can result in decreased DO. Increases in temperature cause decreased solubility of oxygen and increased level of ammonia in water.^19^ The optimum temperature range for milkfish culture is 20 - 43°C.^20^ Increases in temperature lowers the solubility of oxygen, which could cause stress and death at extreme temperatures.^21^

Fish have an average blood pH of 7.4 and a small deviation from this value, between 7.0 and 8.5, is more optimal and conducive to fish life. Fish can become stressed in water with a pH ranging from 4.0 to 6.5 and 9.0 to 11.0, and death is almost certain at a pH less than 4.0 or greater than 11.0.^22^ The pH levels in the present study were within optimum levels.

High levels of ammonia could damage the gills, destroy mucous-producing membranes and cause sub-lethal effects such as reduced growth, poor feed conversion and reduced disease resistance. Fish suffering from ammonia poisoning generally appear sluggish and appear at the water surface gasping for air.^19^ The applied zeolite could reduce the content of ammonia in waters with an efficiency exceeding 83% with an initial content of ammonia ≤ 1.0 ppm.^23^ When BOD levels are high, dissolved oxygen decreases because oxygen that is available in the water is being consumed by the bacteria that can cause fish death.^24^ A BOD level between 3.0 – 6.0 ppm is optimum for normal fish activity.^25^

Copper levels at locations which receive anthropogenic inputs may have high concentrations in water. Mining, leather and leather products, fabricated metal products and electrical equipment are industries with Cu-bearing discharges that contribute to anthropogenic inputs of Cu to surface water.^26^ Since Marilao and Meycauayan rivers are known to be polluted with heavy metals, there is a high probability that the aquaculture sector may be affected by Cu toxicity.

### Fish quality analysis

The feed conversion ratio is a measure of the amount of feed required to produce a kilogram of milkfish.^27^ The lower the FCR value, the less feed needed to produce a kilogram of milkfish. The FCR of fishes is determined by the metabolic capacity of fish to digest a given feed, and is affected by size, diet quality, feed regime, water temperature and fish status.^28^

### Sediment quality analysis

Zeolites are widely used in the aquaculture industry in Southeast Asia and Latin America to improve water and feed quality and to reduce the negative environmental impacts of aquaculture and to improve the quality of cultivated seafood.^23^ A study conducted on Cd content in the rearing water of Mozambique tilapia showed a reduction after 45 days exposure time with removal efficiency of 75% using zeolite at 4 g/L.^29^

Comparing the levels of heavy metals with the baseline assessment, it was observed that some of the ponds had decreased lead levels relative to baseline (before application of zeolite). Chromium levels were less than the limit of detection after three months of culture for the majority of ponds at the Nagbalon site. Application of zeolite may not have decreased the heavy metal content of sediments, but is still a prospect to address heavy metal contamination in sediments through the adsorption of zeolite, making heavy metals unavailable for fishes. Proper and frequent application of zeolite is required to achieve the desired effect of this technology. Toxicity characterization leaching procedure must also be performed to determine if the heavy metals present are highly toxic to fishes. Studies have demonstrated that use of zeolites in fish culture has shown a significant positive effect on fish that consume them, similar to the addition of antibiotics. Zeolites also have the ability to decrease ammonia levels, which could be very toxic for fish, and can lead to decreased growth, even in small concentrations.^30^

Heavy metal removal efficiency from aqueous solutions using zeolite is affected by several factors such as pH, temperature, presence of other contaminants in the treated water, properties of heavy metal ions, pre-treatment applied to zeolite, pore clogging, particle size, and zeolite purity. The removal efficiency of zeolite for other heavy metals such as Cd, Cr, Cu, Ni, Zn, iron (Fe), and Pb were 90, 90, 90, 75, 85, 70, and 95%, respectively.^23^

### Impacts of different remediation strategies

The remediation strategies used in the present study were assessed using various parameters. The results of the present study showed that application of ponds with probiotics had a positive effect on the growth of milkfish for the Nagbalon site. Ponds applied with probiotics had the highest average fish weight for Pond 1 and Pond 2. The FCR of fish was relatively low compared to the control pond. The probiotics helped the milkfish improve their feed digestibility and feed utilization. Application of zeolite in sediments resulted in decreased levels of lead during the culture period. Ponds showed a slightly alkaline pH level throughout the culture period. Despite the short span of the phytoremediation set up, there were substantial changes in water quality. Ammonia levels showed a decreasing trend in the treatment ponds. The levels of ammonia, BOD and COD in the phytoremediation ponds at both sites were relatively lower than the other ponds. Another positive impact brought about by the vetiver grass system is that it served as shade for the fish during high temperatures. This helped to reduce the stress on fish and helped them survive higher temperatures. The FCR of milkfish in the phytoremediation pond was the lowest for the Liputan site. This indicates there was an efficient conversion of feeds to biomass in ponds with vetiver grass.

Table 13 summarizes the cost-effectiveness of the different remediation strategies employed in the present study.

**Table 13:**
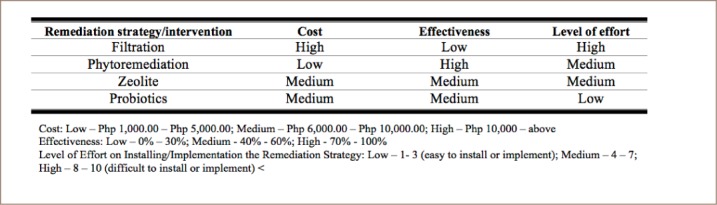
Summary of the Cost-effectiveness of the Different Remediation Strategies

## Conclusions

The environmental quality of aquaculture ponds was assessed to determine the effectiveness of different remediation strategies. The DO level of ponds at both study sites was below the recommended level in the morning and at a supersaturated level in the afternoon. Ammonia and COD exceeded recommended levels. However, these two parameters were much lower in the phytoremediation ponds compared to other pond setups. There was also a decreasing trend observed for ammonia levels in treatment ponds. Small amounts of Cu were detected in pond water. In terms of milkfish growth, the pond with probiotics had the highest average weight at the Nagbalon site, while the phytoremediation pond had the highest rate at Liputan. The percentage survival of milkfish was higher in Liputan than in Nagbalon. Copper, Cr, Pb and Mn were the only heavy metals detected in sediments. Throughout the culture period, there was a decrease in Pb concentration in sediments after the application of zeolite.

The application of probiotics in the present study influenced fish growth, as reflected by milkfish harvested at the Nagbalon site. It also showed lower FCR values compared to other ponds. The probiotics somehow helped the milkfish improve their feed digestibility and utilization. Application of zeolite in sediments resulted in decreased levels of Pb during the culture period. The phytoremediation setup showed a decreased level of some parameters such as ammonia, BOD, and COD. At the Liputan site, the milkfish grown in the phytoremediation pond had the highest growth and better FCR values. Phytoremediation appeared to be the most cost-effective strategy used in the present study.

## Recommendations

To improve the efficiency and effectiveness of the different remediation strategies, improvements are needed in the engineering design of the vetiver grass system (phytoremediation) and proper application of probiotics and zeolite. Further experiment cycles are needed to validate the results of the present study along with continuous research and development of these strategies to establish their efficiency and cost-effectiveness. Controls with probiotics and zeolite are needed to delineate their effect on COD, BOD, pH and metal removal.
